# The NaHCO_3_-Responsive Phenotype in Methicillin-Resistant Staphylococcus aureus (MRSA) Is Influenced by *mecA* Genotype

**DOI:** 10.1128/aac.00252-22

**Published:** 2022-05-16

**Authors:** Selvi C. Ersoy, Adhar C. Manna, Richard A. Proctor, Henry F. Chambers, Ewan M. Harrison, Arnold S. Bayer, Ambrose Cheung

**Affiliations:** a The Lundquist Institute, Torrance, California, USA; b Department of Microbiology & Immunology, Geisel School of Medicine at Dartmouth, Hanover, New Hampshire, USA; c University of Wisconsin School of Medicine and Public Health, Madison, Wisconsin, USA; d UCSF School of Medicine, San Francisco, California, USA; e Wellcome Sanger Institute, Hinxton, United Kingdom; f Department of Medicine, University of Cambridge, Cambridge, United Kingdom; g Department of Public Health and Primary Care, University of Cambridge, Cambridge, United Kingdom; h The Geffen School of Medicine, University of California, Los Angeles, California, USA

**Keywords:** Methicillin-resistant *Staphylococcus aureus* (MRSA), β-lactam, sodium bicarbonate (NaHCO_3_), *mecA*, penicillin-binding protein 2a (PBP2a)

## Abstract

Methicillin-resistant Staphylococcus aureus (MRSA) strains are a leading cause of many invasive clinical syndromes, and pose treatment difficulties due to their *in vitro* resistance to most β-lactams on standard laboratory testing. A novel phenotype frequently identified in MRSA strains, termed ‘NaHCO_3_-responsiveness’, is a property whereby strains are susceptible *in vitro* to many β-lactams in the presence of NaHCO_3_. Specific *mecA* genotypes, repression of *mecA*/PBP2a expression and perturbed maturation of PBP2a by NaHCO_3_ have all been associated with this phenotype. The aim of this study was to define the relationship between specific *mecA* genotypes and PBP2a substitutions, on the one hand, with NaHCO_3_-responsiveness *in vitro*. Mutations were made in the *mecA* ribosomal binding site (RBS -7) and at amino acid position 246 of its coding region in parental strains MW2 (NaHCO_3_-responsive) and C36 (NaHCO_3_- nonresponsive) to generate ‘swap’ variants, each harboring the other’s *mecA*-RBS/coding region genotypes. Successful swaps were confirmed by both sequencing, as well as predicted swap of *in vitro* penicillin-clavulanate susceptibility phenotypes. MW2 swap variants harboring the nonresponsive *mecA* genotypes became NaHCO_3_-nonresponsive (resistant to the β-lactam, oxacillin [OXA]), in the presence of NaHCO_3_. Moreover, these swap variants had lost NaHCO_3_-mediated repression of *mecA*/PBP2a expression. In contrast, C36 swap variants harboring the NaHCO_3_-responsive *mecA* genotypes remained NaHCO_3_-nonresponsive phenotypically, and still exhibited nonrepressible *mecA*/PBP2a expression. These data demonstrate that in addition to the *mecA* genotype, NaHCO_3_-responsiveness may also depend on strain-specific genetic backgrounds.

## INTRODUCTION

Methicillin-resistant Staphylococcus aureus (MRSA) is a leading cause of invasive bacterial infections in both children and adults worldwide (1–3). MRSA infections cause a wide variety of syndromes, including skin and soft tissue infections, bacteremia, and endocarditis (3, 4). Compared to infection with methicillin-susceptible S. aureus (MSSA), MRSA are more difficult to treat due to their resistance to nearly all β-lactams (the treatment of choice for MSSA [3, 5–7]), leaving limited therapeutic alternatives.

Recently, efforts have been made to more accurately determine *in vitro* antimicrobial susceptibility profiles under conditions more relevant to host physiologic microenvironments, including for MRSA (8–14). These investigations have led to the discovery of a novel phenotype in a rather large proportion of MRSA strains, termed “NaHCO_3_-responsiveness” (9, 10, 15). NaHCO_3_-responsive strains display a substantial (≥4-fold) reduction in their *in vitro* MICs for certain β-lactams (usually into the ‘susceptible’ MIC range) when grown in the presence of NaHCO_3_ (10, 15). Moreover, NaHCO_3_-responsive MRSA strains are effectively cleared by β-lactam therapy in both *ex vivo* and *in vivo* experimental endocarditis models (10).

This phenotype is multifactorial, but features perturbations in: (i) expression of *mecA*, which encodes the alternative penicillin-binding protein (PBP) 2a, the primary determinant of β-lactam resistance in S. aureus (16–19); and (ii) other genes necessary for the proper functioning of PBP2a in carrying out cell wall biosynthesis (10, 20, 21). PBP2a functions via allosteric regulation of the active binding site for peptidoglycan precursers, which remains “closed” in the presence of traditional β-lactams (22–24). In contrast, newer generation cephalosporins (ceftaroline; ceftabiprole) are able to open such sites (25, 26).

Recently, another unique β-lactam susceptibility phenotype in MRSA was identified by Harrison et al., wherein strains possessing distinct *mecA* genotypes were found to be either ‘susceptible’ or ‘resistant’ to a combination of β-lactam/β-lactamase inhibitors (27). These investigations identified specific regions within *mecA* of particular importance to this phenotype, including both the upstream -7 site, corresponding to the *mecA* promoter/ribosomal binding site (RBS) region (28), as well as the 246^th^ amino acid position of PBP2a (27). Alteration of the promoter/RBS sequence was found to alter expression of *mecA*, while substitution at the 246^th^ amino acid position was found to alter penicillin binding to PBP2a in the presence of clavulanate (27). Thus, specific *mecA* genotypes, with respect to these two loci, have been dubbed ‘susceptible’ or ‘resistant’ in relation to the ability of β-lactam/β-lactamase inhibitors to inhibit such strains (27).

The aims of the current study were to determine the impacts of particular ‘susceptible’ and ‘resistant’ *mecA* genotypes (as per Harrison et al. [27]), on the one hand, with the NaHCO_3_-responsiveness phenotype, on the other hand. To this end, we constructed isogenic mutants with various ‘susceptible’ and ‘resistant’ *mecA* genotypes, as defined above (27), in prototype NaHCO_3_-responsive and nonresponsive MRSA strain backgrounds. These mutant strains were then assessed for both ‘swap’ of their susceptibility to β-lactams/β-lactamase inhibitors, as well of their NaHCO_3_-responsiveness phenotypes. Additionally, the impact of these mutational swaps on *mecA* transcription, translation, and PBP2a protein production/localization was determined.

## RESULTS

### Impact of *mecA* alleles on susceptibility to β-lactams.

To determine the impact of altered *mecA* RBS and/or coding sequences on susceptibility to β-lactams, isogenic mutants were constructed in the MW2 (NaHCO_3_-responsive) and C36 (nonresponsive) parental backgrounds harboring chromosomal alterations at the *mecA* RBS -7 site and/or the 246^th^ amino acid position in PBP2a. The NaHCO_3_-responsiveness phenotype is defined by ≥ 4-fold increased susceptibility to β-lactam antibiotic in the presence of NaHCO_3_ (44 mM) compared to its absence (10). As above, the specific point mutations introduced were originally designated being determinative of “susceptibility” or “resistance” to penicillin in the presence of clavulanic acid (27). Specifically, strains harboring the RBS -7T allele (with either 246E or 246G amino acid) were determined to be susceptible to the combination of penicillin (PEN) plus clavulanic acid (CLAV); strains harboring the RBS -7G allele (with either 246E or 246G amino acid) were resistant to this β-lactam/β-lactamase combination (27). Assessment of the impact of these genetic alterations in the MW2 and C36 strain backgrounds revealed that swap mutants harboring the RBS -7G allele were more resistant to penicillin plus clavulanic acid by Etest than strains harboring the RBS -7T allele in both backgrounds (Table 1), confirming prior reports (27–29).

**TABLE 1:** Summary of penicillin + clavulanate and oxacillin susceptibility, and *mecA*/PBP2a expression for MW2 and C36 parental strains and *mecA* swap mutants

	Allele		Oxacillin MIC (μg/mL)			PEN + CLAV MIC (μg/mL) (S, I, or R^*b*^)	NaHCO_3_ impact on:	
Strain	RBS	246AA	CA-MHB^*a*^	CA-MHB tris	CA-MHB tris 44 mM NaHCO_3_		*mecA* gene expression^*c*^	PBP2a protein production^*d*^
MW2 parent	T	G	32	64	2	0.38 (S)	Decrease	Decrease
ALC9188	T	E	4	8	2	0.25 (S)	Decrease	Decrease
ALC9200	G	G	64	128	64	12 (R)	No decrease	No decrease
ALC9196	G	E	32	64	32	2 (I)	No decrease	No decrease
C36 parent	G	G	512	1024	1024	12 (R)	No decrease	No decrease
ALC9259	G	E	64	64	512	2 (I)	No decrease	No decrease
ALC9268	T	G	64	64	256	1.5 (S)	No decrease	No decrease
ALC9322	T	E	64	64	256	0.25 (S)	No decrease	No decrease

*^a^*CA-MHB, cation-adjusted Mueller Hinton Broth.

*^b^*Susceptible (S) PEN + CLAV MIC is an MIC of < 2 μg/mL by Etest on ISA media; intermediate (I) PEN + CLAV MIC is an MIC = 2 μg/mL by Etest on ISA media; resistant (R) PEN + CLAV MIC is an MIC >2 μg/mL by Etest on ISA media.

*^c^*Exposure to 44 mM NaHCO_3_ + 1/2× MIC oxacillin results in decreased, increased, or no change in *mecA* expression compared to exposure to 1/2× MIC oxacillin alone by qRT-PCR.

*^d^*Exposure to 44 mM NaHCO_3_ + 1/2× MIC oxacillin results in decreased PBP2a protein production compared to exposure to 1/2× MIC oxacillin alone by Western blotting.

The parental responsive MW2 and nonresponsive C36 stains (10, 15, 30) and their swap constructs were then assessed for susceptibility to the anti-staphylococcal β-lactam, oxacillin (OXA), in the presence or absence of NaHCO_3_. Upon minimum inhibitory concentration (MIC) assessment of the MW2 parent and its *mecA* swap mutants, alteration of the RBS -7 site from T-to-G eliminated the NaHCO_3_-responsiveness phenotype to OXA in this background, such that mutants harboring the RBS -7G allele (ALC9200 and ALC9196) were highly resistant to OXA in the presence of NaHCO_3_ (Table 1).

Despite the ability of the RBS -7G allele to reverse the NaHCO_3_-responsive phenotype to OXA in the MW2 background, introduction of the RBS -7T allele into the C36 nonresponsive parental background (ALC9268 and ALC9322) did not establish the responsive phenotype to OXA (Table 1). The ability of the RBS site mutation to alter penicillin/clavulanic acid susceptibility, but not NaHCO_3_-mediated β-lactam susceptibility in the nonresponsive strain background, indicates that additional elements are involved in the NaHCO_3_-responsive phenotype besides these specific *mecA* RBS genotypes.

### Effect of *mecA* alleles on *mecA* transcription, translation, and PBP2a production/localization.

**FIG 1:**
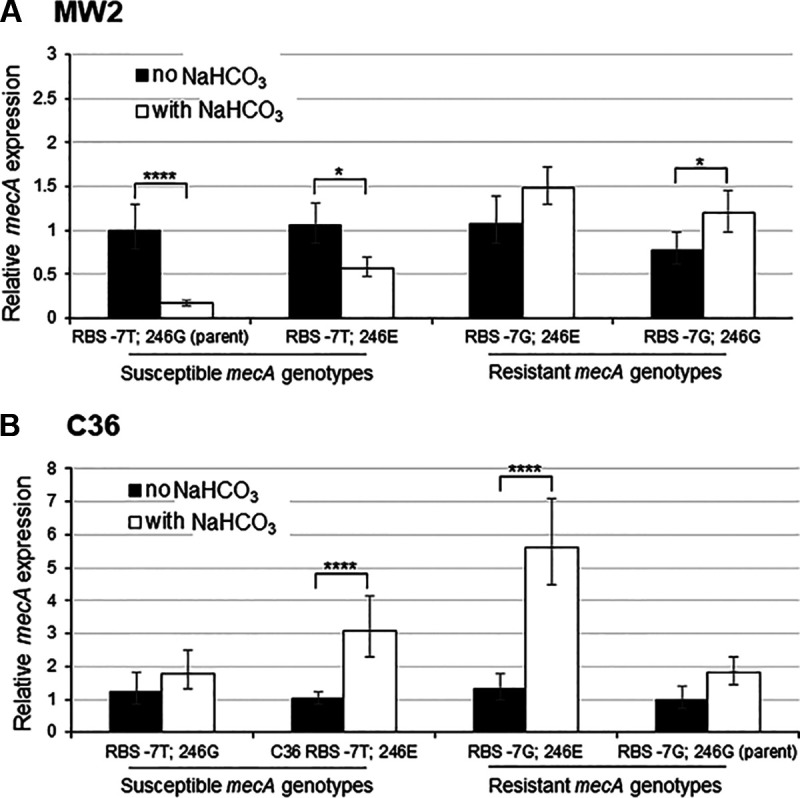
Expression of *mecA* in (A) NaHCO_3_-responsive strain MW2 background and (B) nonresponsive strain C36 background. Gene expression data were obtained by qRT-PCR of RNA from MW2 and C36 *mecA* swap constructs grown in cation-adjusted Mueller-Hinton Broth (CA-MHB) + Tris + 1/2× MIC OXA (no NaHCO_3_) and + 44 mM NaHCO_3_ (with NaHCO_3_). 2% NaCl was included in all growth media in which OXA was also included. For each strain background, *mecA* expression was normalized to the value obtained in CA-MHB Tris + 1/2× MIC OXA for the parental strain (MW2 or C36), with this value set equal to 1.0. Statistics were determined by a Student's *t* test; *, *P* < 0.05; ****, *P* < 0.0001.

To assess the impact of alterations to the RBS/promoter region of *mecA* on its transcription, *mecA* gene expression after OXA induction was quantified by qRT-PCR, in the presence and absence of NaHCO_3_ for MW2 and C36 parental strains, as well as their respective *mecA* variant swap constructs. Although the *mecA* start codon upstream -7 site has been identified as part of the putative ribosomal-binding site (RBS), point mutations at this location have been reported to impact on *mecA* transcription (27, 28). We previously demonstrated that NaHCO_3_ repressed expression of *mecA* specifically in NaHCO_3_-responsive MRSA strains, but not in nonresponsive strains (10, 20). When *mecA* transcription was assessed in the absence of NaHCO_3_, similar expression levels were observed for both parental strains and their swap constructs (Fig. 1A and B). However, in the presence of NaHCO_3_, *mecA* transcription was highly repressed compared to expression in the absence of NaHCO_3_ for MW2 parent and mutants harboring the RBS -7T allele; in contrast, *mecA* expression was not repressible by NaHCO_3_ for MW2 swap mutants harboring the RBS -7G allele (Fig. 1A). In the C36 strain background, introduction of the RBS -7T allele did not result in NaHCO_3_-mediated repression of *mecA* (Fig. 1B).

**FIG 2:**
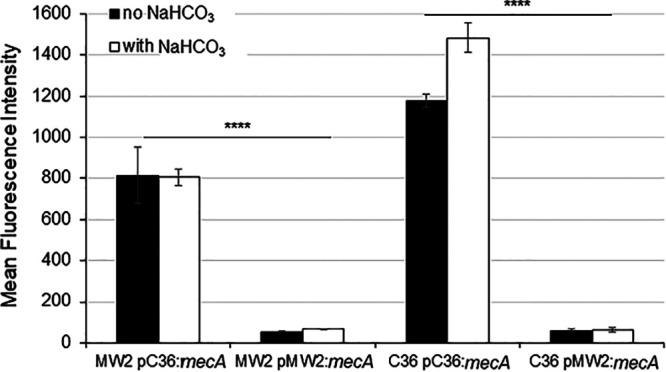
Translational efficiency of native MW2 and C36 *mecA* RBS/promoter sequences. Translation was assessed by flow cytometry using strains harboring promoter-GFP fusions for the MW2 (RBS -7T) and C36 (RBS -7G) RBS/promoter regions. Cells were grown in cation-adjusted Mueller-Hinton Broth (CA-MHB) + Tris + 1/2× MIC OXA (no NaHCO_3_) and + 44 mM NaHCO_3_ (with NaHCO_3_) for 3 h before being assessed for GFP fluorescence by flow cytometry. 2% NaCl was included in all growth media in which OXA was also included. Statistics were determined by a Student's *t* test; ****, *P* < 0.0001.

To determine the influence of altering the RBS -7 site on *mecA* translation in MW2 and C36 strain backgrounds, translational GFP reporter fusion constructs were generated for the native MW2 and C36 *mecA* promoter regions, and swap-transformed back into MW2 and C36. Translational efficiency of each GFP fusion construct was assessed by flow cytometry, in the presence and absence of NaHCO_3_. Interestingly, unlike the transcriptional data, alteration of the RBS -7 site had substantial impact on the translational efficiency of each construct, regardless of the strain background. In both MW2 and C36, strains harboring the RBS -7G construct (C36 native *mecA* promoter sequence) had significantly more GFP production than those harboring the RBS -7T construct (MW2 native *mecA* promoter sequence) (Fig. 2). Of note, growth in NaHCO_3_-containing media did not reduce the translational efficiency of the RBS -7T fusion, and slightly enhanced translation of the RBS -7G fusion in the C36 background (Fig. 2). Taken together with the *mecA* expression data, this indicates that NaHCO_3_ may be specifically impacting *mecA* expression more preferentially at the transcriptional, rather than at the translational level, in responsive strains.

**FIG 3:**
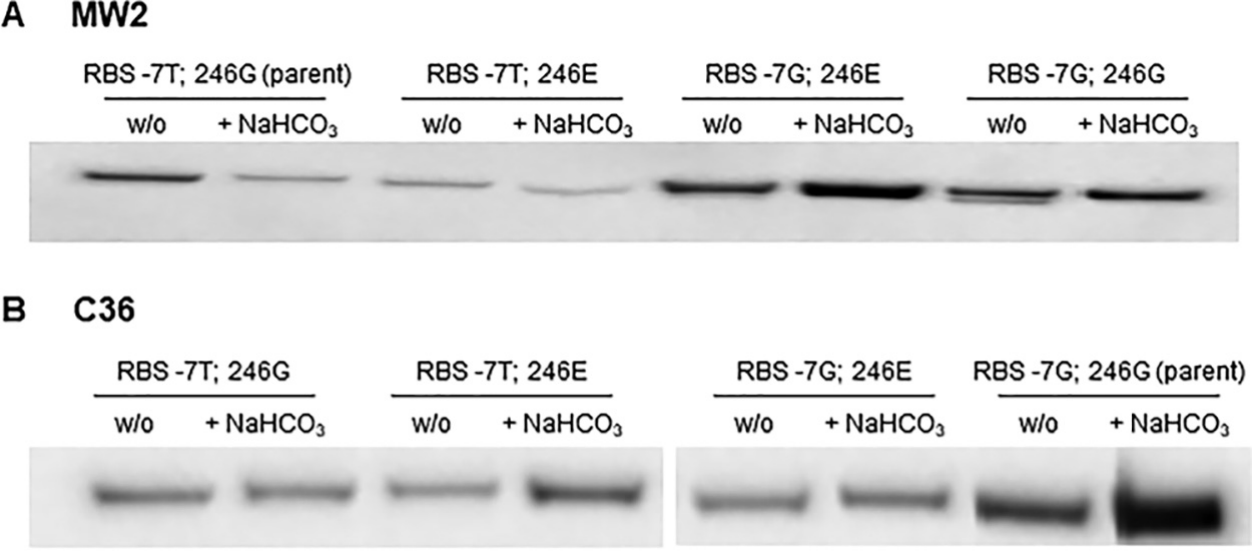
PBP2a protein production and membrane localization in (A) NaHCO_3_-responsive strain MW2 background and (B) nonresponsive strain C36 background. Protein expression and localization were assessed by Western blotting of the membrane protein fraction from MW2 and C36 *mecA* swap constructs grown in cation-adjusted Mueller-Hinton Broth (CA-MHB) + Tris + 1/2× MIC OXA (w/o) or CA-MHB Tris + 1/2 × MIC OXA + 44 mM NaHCO_3_ (+ NaHCO_3_). 2% NaCl was included in all growth media in which OXA was also included.

Finally, to determine the overall impact of specific *mecA* alleles on PBP2a production and membrane localization, Western blotting was performed on the membrane protein fraction of MW2 and C36 *mecA* mutant constructs grown in the presence and absence of NaHCO_3_. In the MW2 background, strains harboring the RBS -7T variant had reduced amounts of membrane-localized PBP2a when grown in the presence versus absence of NaHCO_3_ (Fig. 3A), corresponding to the observed repression of *mecA* transcription in the presence of NaHCO_3_ observed in these strains (Fig. 1A). MW2 strains harboring the RBS -7G allele had overall increased membrane-associated PBP2a in both the presence and absence of NaHCO_3_ compared to strains harboring the RBS -7T allele (Fig. 3A); this outcome corresponded to the increased translational efficiency of this sequence variant (Fig. 2). In the C36 background, all strains had similar or increased levels of membrane PBP2a in the presence versus absence of NaHCO_3_ (Fig. 3B), and no consistent pattern was seen between the RBS -7T and -7G variant.

## DISCUSSION

The recent discoveries of the impact of specific *mecA* alleles on β-lactam susceptibility phenotypes appear to reveal novel paradigms for further understanding of β-lactam resistance in MRSA. Thus, mutations within the *mecA* promoter at the -7 site (a part of the RBS) have now been linked to both altered susceptibility to a combination of β-lactam/β-lactamase inhibitors, as well as to the NaHCO_3_-β-lactam-responsive phenotype (27, 30).

A major change following the swap-in at the RBS -7 site into the NaHCO_3_-responsive strain, MW2, appeared to be at the *mecA* transcriptional level. Previously, NaHCO_3_ was shown to repress expression of *mecA* specifically in NaHCO_3_-responsive strains, such as MW2 (10, 20). Herein, we observed that MW2 harboring the native RBS -7T allele had the predicted NaHCO_3_-repressible *mecA* expression outcome; in contrast, *mecA* expression in swap mutants harboring the RBS -7G allele was either not represssed or enhanced by NaHCO_3_. Interestingly, *mecA* expression in the absence of NaHCO_3_ was similar for all mutant strains within a given strain background (MW2 and C36). This finding is contrary to that identified by Harrison et al. (27) and Chen et al. (28), whereby alteration of the RBS -7 site from T-to-G resulted in enhanced *mecA* transcription under OXA-induction conditions (27). However, it should also be underscored that Harrison et al. and others reported individual strain-to-strain variations in the latter relationship (27, 29); this seems to indicate that other strain-specific factors or environmental cues (e.g., in growth media) influence how *mecA* promoter mutations affect *mecA* transcription. Lastly, in our studies, alteration of the RBS -7 from T-to-G did result in a substantial increase in PBP2a translation in both strain backgrounds, as well as PBP2a membrane protein production in the MW2 background (and in one of the two swaps in C36); these latter data are similar to a previous report (28), and more consistent with this site’s identified role within the RBS.

Based on these current data-sets, one likely mechanism involved in reversing NaHCO_3_ responsiveness in MW2 swap strains may be a de-repression of *mecA* expression by NaHCO_3_ in swap strains harboring the RBS -7G allele. Such an event would result in increased PBP2a production and higher OXA MICs in the presence of NaHCO_3_. Interestingly, other studies have found that alteration of *mecA*/PBP2a expression does not necessarily directly correlate to β-lactam MIC levels (28, 31–33). However, our data reveal a more direct correlation between PBP2a protein levels and MICs for all strains and conditions observed in this study. This was particularly noted for the C36 parental strain, which produced substantially more PBP2a than the other three C36 *mecA* swap variant strains; their MICs in media with and without NaHCO_3_ were 2- to 8-fold greater than the other *mecA* variants within this background. These data indicate that strain-specific factors influence PBP2a production, dictating the specific MIC for that strain. Of note, alteration of the RBS -7 G-to-T sequence in the C36 background did not result in NaHCO_3_-mediated repression of *mecA*/PBP2a. Although this observation explains why this genetic alteration did not evoke a NaHCO_3_-responsiveness phenotype in this strain background, it raises the notion that other factors, besides the *mecA* RBS sequence, must be required to stimulate NaHCO_3_-mediated *mecA* gene repression to subsequently yield a NaHCO_3_-responsive phenotype.

Taken together, these data support a primary role of the RBS -7 site as a mediator of *mecA* transcription and PBP2a translation in the NaHCO_3_-responsive strain, MW2. Although the RBS -7 site is clearly important for the maintenance of the responsive phenotype, it does not appear to be sufficient to generate this phenotype in a nonresponsive strain background. More work must be undertaken in additional MRSA strain backgrounds to elucidate the genetic and molecular mechanisms required to generate the NaHCO_3_-responsive phenotype, and to understand their role in the context of *mecA* transcriptional regulation.

One possible explanation for the differential ability of NaHCO_3_ to repress *mecA* transcription in the MW2 versus C36 background strains may be intrinsic differences in the native SCC*mec* cassettes in each strain background. NaHCO_3_-responsive strain, MW2, possesses SCC*mec* type IV, whereas the nonresponsive strain C36 possesses SCC*mec* type II (30). Although linkage between specific SCC*mec* types and the NaHCO_3_-responsive versus-nonresponsive phenotypes has not been established (24), the underlying genetic differences between these two cassettes may contribute to the ability of specific *mecA* genotypes to confer NaHCO_3_-responsiveness. Specifically, the SCC*mec* type IV cassette has truncations within *mecI*-*mecR1*, encoding the *mecA* inhibitor gene (*mecI*) and the cognate response regulator (*mecR1*), rendering these genes nonfunctional; in contrast, the SCC*mec* type II cassette has an intact and functional *mecA-mecI*-*mecR1* regulator*y* axis (34–36). The lack of an intact *mecI*-*mecR1* system in the responsive strain, MW2, indicates *mecA* expression is probably regulated by β-lactamase-regulatory elements, *blaI*-*blaR1*, and possibly other, as yet undefined regulatory systems. Alternatively, *mecA* expression in strains within the C36 genetic background may be more tightly regulated by their intact *mecI*-*mecR1* system. It might be speculated that in the presence of functional *mecI*-*mecR1*, alteration of the RBS -7G-to-T is insufficient to allow for NaHCO_3_-mediated repression of *mecA* transcription. Conversely, in the absence of intact *mecI*-*mecR1*, the RBS -7T allele can confer NaHCO_3_-mediated repression of *mecA*. Further studies are being carried out to explore these possibilities.

Finally, it should be noted that some of the disparity between the ‘swap’ results, in terms of penicillin-clavulanate versus OXA-NaHCO_3_-responsiveness MIC metrics may have been influenced by the differential PBP2a binding affinity of penicillin versus OXA, which differ by ~20-fold (18).

Of note, we did not observe any impacts of alteration of the 246^th^ amino acid position alone on the NaHCO_3_-responsive phenotype in either strain background, despite this mutation being associated with altered susceptibility to β-lactam/β-lactamase inhibitor combinations (27). Interestingly, in a separate study, we did observe that purified PBP2a 246E and 246G protein variants had differential binding affinities for Bocillin-FL in the presence of NaHCO_3_ (37). These latter data imply that the specific PBP2a protein variant present in a strain background may alter the binding affinity of a given strain for β-lactams in the presence of NaHCO_3_, although on its own, this polymorphism is not sufficient to alter the NaHCO_3_-responsive phenotype of a given strain. We hypothesized that this difference in β-lactam binding affinity between the two PBP2a variants in the presence of NaHCO_3_ may be due to an altered NaHCO_3_ buffering capacity between the glutamic acid (246E) and glycine (246G) residues present in their specific allosteric binding domains (22).

Overall, this study elucidates the impact of *mecA* sequence polymorphisms on the NaHCO_3_-responsive phenotype in MRSA, and sheds further light on the complex regulation of methicillin resistance in S. aureus.

## MATERIALS AND METHODS

### Strains, media, and growth conditions.

The primary parental strains utilized in this study were the prototypical and well-characterized NaHCO_3_-responsive strain, MW2 (10, 20, 30, 38) and the nonresponsive strain, C36 (15, 30) (Table 2). Strains MW2 and C36 have previously been identified as having either the “susceptible 2” or “resistant 2” *mecA* genotypes, respectively (30), as defined by Harrison et al. (27). Strains were stored at −80°C and isolated on tryptic soy agar (TSA) at 37°C in ambient air when ready for use. All liquid cultures were grown at 37°C in ambient air with aeration.

**TABLE 2:** Strains and plasmids used in this study

Strains or plasmids	Relevant features	Reference
MW2 (parent)	*mecA* genotype: RBS -7T, 246G; CC type: 1; *spa* type: t128; SCC*mec* type: IV	(30, 38)
ALC9188 (MW2 derivative)	*mecA* genotype: RBS -7T, 246E	This study
ALC9200 (MW2 derivative)	*mecA* genotype: RBS -7G, 246G	This study
ALC9196 (MW2 derivative)	*mecA* genotype: RBS -7G, 246E	This study

C36 (parent)	*mecA* genotype: RBS -7G, 246G; CC type: 5; *spa* type: t002; SCC*mec* type: II	(15, 30)
ALC9259 (C36 derivative)	*mecA* genotype: RBS -7G, 246E *erm^R^*	This study
ALC9268 (C36 derivative)	*mecA* genotype: RBS -7T, 246G *erm^R^*	This study
ALC9322 (C36 derivative)	*mecA* genotype: RBS -7T, 246E *erm^R^*	This study

Plasmids ALC9332 (MW2 derivative)	pALC1484 pC36*mecA*::*gfp_uvr_ chlor^R^*	This study
ALC9334 (MW2 derivative)	pALC1484 pMW2*mecA*::*gfp_uvr_ chlor^R^*	This study
ALC9329 (C36 derivative)	pALC1484 pC36*mecA*::*gfp_uvr_ chlor^R^*	This study
ALC9330 (C36 derivative)	pALC1484 pMW2*mecA*::*gfp_uvr_ chlor^R^*	This study
		
pMAD pMAD-X	β-gal, *erm^R^* β-gal, *chlor^R^, modified pMAD* with *cat* gene by removing *erm* gene	(40) This study
pALC1484	Promoter-less *gfp_uvr_*, *chlor^R^*	(42)

For penicillin Etest susceptibility testing of penicillin-clavulanate combinations, Iso-Sensitest Agar (ISA, Oxoid) was prepared as per manufacturer’s instructions, with or without 15 μg/mL clavulanic acid (Sigma-Aldrich; see below for further details). For broth MIC testing, RNA isolation/gene expression studies, GFP reporter assays, and Western blotting, strains were cultured in cation-adjusted Mueller-Hinton Broth (CA-MHB, Difco, Beckton-Dickinson) or CA-MHB buffered with 100 mM Tris (CA-MHB Tris) to maintain pH 7.3 ± 0.1, with or without 44 mM NaHCO_3_. Where indicated, 1/2× MIC of OXA (Table 1) was also added to the growth media to stimulate *mecA* expression. In all experiments in which OXA was added to the growth medium, 2% NaCl was also included.

### Construction of various S. aureus mutant and reporter strains.

To determine the contribution of the -7-nucleotide position (AGGAG**G****/T**) (corresponding to the ribosome-binding site, RBS [28],) from the ATG start codon or the amino acid position at 246-residue (Glu/Gly) of the *mecA* gene in NaHCO_3_-responsive or nonresponsive S. aureus strains, we have constructed chromosomal point mutations of the *mecA* region in S. aureus strains MW2 and C36 (erm^R^) using routine procedures as described (39). To construct mutations or interchange the region, a 3.2 kb DNA fragment was amplified that contained the intact *mecA* and *mecR* genes by PCR using primers flanking with BamHI site at both ends (Table S1). The DNA fragment was cloned into a temperature-sensitive shuttle vector pMAD (β-gal, erm^R^) (40) or pMAD-X (β-gal, modified with chlor^R^ by removing erm^R^), and then selected in E. coli IM08B (41) for the correct construct. To construct point mutations at the RBS or at the 246th residue position, site-specific mutagenesis was performed with pMAD constructs as the template and various mutagenized primers using a PCR based method with *PFU* Taq-polymerase (Phusion, Thermo Scientific). After verification by restriction digestion and DNA sequencing, interchanged or point mutation constructs were introduced into various strains by electroporation and selected on erythromycin or chloramphenicol and X-Gal-containing plates for blue colonies at 30°C. Plasmid DNA was isolated and digested with BamHI for the authentication of the presence of DNA fragment in the respective constructs in the strains. The construction of chromosomal mutations in the respective strain by recombination or two-point crossover was performed by routine procedure as described previously (39). Briefly, two-point crossover of the *mecA-mecR* region was performed by temperature shift by growing at strains 43°C with erythromycin or chloramphenicol followed by 30°C subculturing without any antibiotics. Cells were plated with and without erythromycin or chloramphenicol in the presence of X-Gal (40 μg/mL) for selection and incubated at 37°C. White/non-blue colonies were cross-streaked to select erm^S^ (MW2) or chlor^s^ (C36) colonies for the potential two-point crossover clones or mutants. The mutants were verified by chromosomal PCR and DNA sequencing of the PCR product.

To determine if AGGAG**G** in C36 (nonresponsive) or AGGAG**T** in MW2 (NaHCO_3_-responsive) RBS sequence variations have any role in the NaHCO_3_-responsiveness phenotype, translational fusions were constructed for these two-promoter regions. The 95-bp intergenic promoter region of the *mecA-mecR* genes was cloned into a promoter-less *gfp_uvr_* reporter shuttle plasmid, pALC1484, at EcoRI and XbaI sites (Fig. S1) (42). Final constructs were verified by DNA sequencing and mobilized into MW2 and C36 strains.

### Etest and broth microdilution susceptibility testing.

E-testing was performed on iso‐sensitest agar (ISA) with or without 15 μg/mL clavulanic acid as previously described (27). Briefly, cells were grown overnight in 1 mL of tryptic soy broth (TSB), then diluted to 1 × 10^8^ CFU/mL in phosphate-buffered saline (PBS) and plated via the Kirby-Bauer inoculation method (43). Etest strips containing benzylpenicillin (bioMérieux) were placed on the inoculated plates, and plates were incubated overnight at 37°C in ambient air. Broth microdilution MICs were performed according to CLSI guidelines as previously described (10, 44, 45). Briefly, cells were grown overnight in the indicated media condition (CA-MHB, CA-MHB Tris, or CA-MHB Tris + 44 mM NaHCO_3_), then diluted into the same media, containing 2% NaCl, with 2-fold serial dilutions of OXA (Sigma-Aldrich) at a final cell concentration of 5 × 10^5^ CFU/mL. Plates were incubated overnight at 37°C in ambient air and the MIC was read as the first well in which visual turbidity was reduced compared to the no drug control well.

### RNA isolation and qRT-PCR analysis of *mecA* gene expression.

RNA was isolated from stationary-phase cells grown in CA-MHB Tris ± 44 mM NaHCO_3_ with 1/2× MIC OXA and 2% NaCl as previously described (10). RNA was released from cell pellets by FastPrep disruption (FP120, Thermo Savant) in Lysing Matrix B tubes (MP Biomedicals) and isolated by column purification (Qiagen). RNA samples were subjected to Turbo DNase treatment (Turbo DNA-free, Invitrogen, Thermo Fisher Scientific) and reverse transcribed to generate a cDNA library (Superscript IV, Invitrogen, Thermo Fisher Scientific). The *mecA* gene transcript was detected by qPCR (StepOne, Applied Biosystems) using primers listed in Table S1. *gyrB* was used as a housekeeping gene to normalize transcript quantities. Relative gene expression was calculated by the ΔΔC_T_ method on biological replicates performed in triplicate in at least two independent runs.

### Assessment of *mecA* promoter-GFP fusions by flow cytometry.

To determine the activity of native MW2 and C36 promoters, GFP production in the reporter strain constructs was measured by flow cytometry. Reporter strains containing the pALC1484 plasmid (Table 2), were grown overnight in CA-MHB Tris ± 44 mM NaHCO_3_, then subcultured into the same media containing 1/2× MIC oxacillin and 2% NaCl and grown for 3 h to reach log phase. Log phase cells were then diluted 1:10 into PBS and assayed for GFP production by flow cytometry with FACScalibur (Becton, Dickinson). The mean fluorescence intensity (MFI) of each sample was calculated with FlowJo software (version 10.8), using data from the FL1-H channel, and expressed as relative fluorescent units/cell population (10,000 cells). All samples were performed in triplicate on two separate days.

### Membrane protein extraction and Western blotting.

Cells were grown to stationary-phase in CA-MHB Tris ± 44 mM NaHCO_3_ with 1/2× MIC oxacillin and 2% NaCl, pelleted, and incubated with DNase (Ambion, Invitrogen), RNase (Thermo Fisher Scientific), and Halt Protease Inhibitor Cocktail (Thermo Fisher Scientific) for 30 min at 37°C and then 15 min at 4°C as previously described (20). The cells were disrupted with glass beads by FastPrep agitation (FP120, Thermo Savant), and centrifuged for 10 min at 4°C and 15,000 RPM to clarify the suspension. The membrane protein fraction was collected from the supernatant by centrifugation for 2 h at 4°C and 15,000 RPM and resuspended in PBS containing Halt Protease Inhibitor. Membrane protein concentration was quantified by Bradford protein assay; 40 μg of membrane proteins were separated on a 4–12% Bis-Tris gel (Invitrogen), run with MES buffer, and blotted onto a nitrocellulose membrane (Amersham). Total protein loading was confirmed by staining with 0.25% ponceau (Fig. S2). The membrane was blocked with 10% dry milk in Tris Buffered Saline with Tween (TBST). PBP2a was probed with a chicken anti-PBP2a antibody (RayBiotech) diluted 1:2500 and detected with an anti-chicken IgY cross-absorbed secondary antibody, HRP (Thermo Fisher Scientific) diluted 1:5000. Labeled proteins were imaged using a c400 imager (Azure Biosystems).
