# Supplementary material for: The NaHCO3-Responsive Phenotype in Methicillin-Resistant Staphylococcus aureus (MRSA) Is Influenced by mecA Genotype
Source: Antimicrob Agents Chemother. 2022 May 16;66(6):e00252-22. doi: 10.1128/aac.00252-22 (PMC9211399; doi:10.1128/aac.00252-22)
Supplement: Supplemental file 1 — Tables S1 and S2 and Fig. S1 to S6. Download aac.00252-22-s0001.pdf, PDF file, 0.3 MB [file aac.00252-22-s0001.pdf]

Supplemental Figure S1

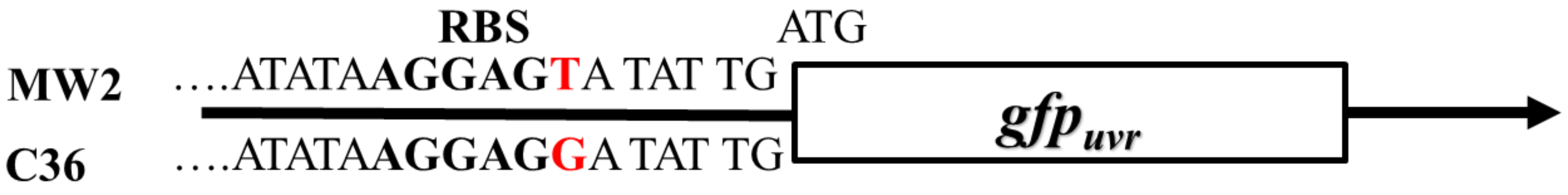

Figure S1. Schematic of translational reporter fusion of MW2 and C36 upstream *mecA* region and *gfp*$_{uvr}$ reporter.

Supplemental Figure S2

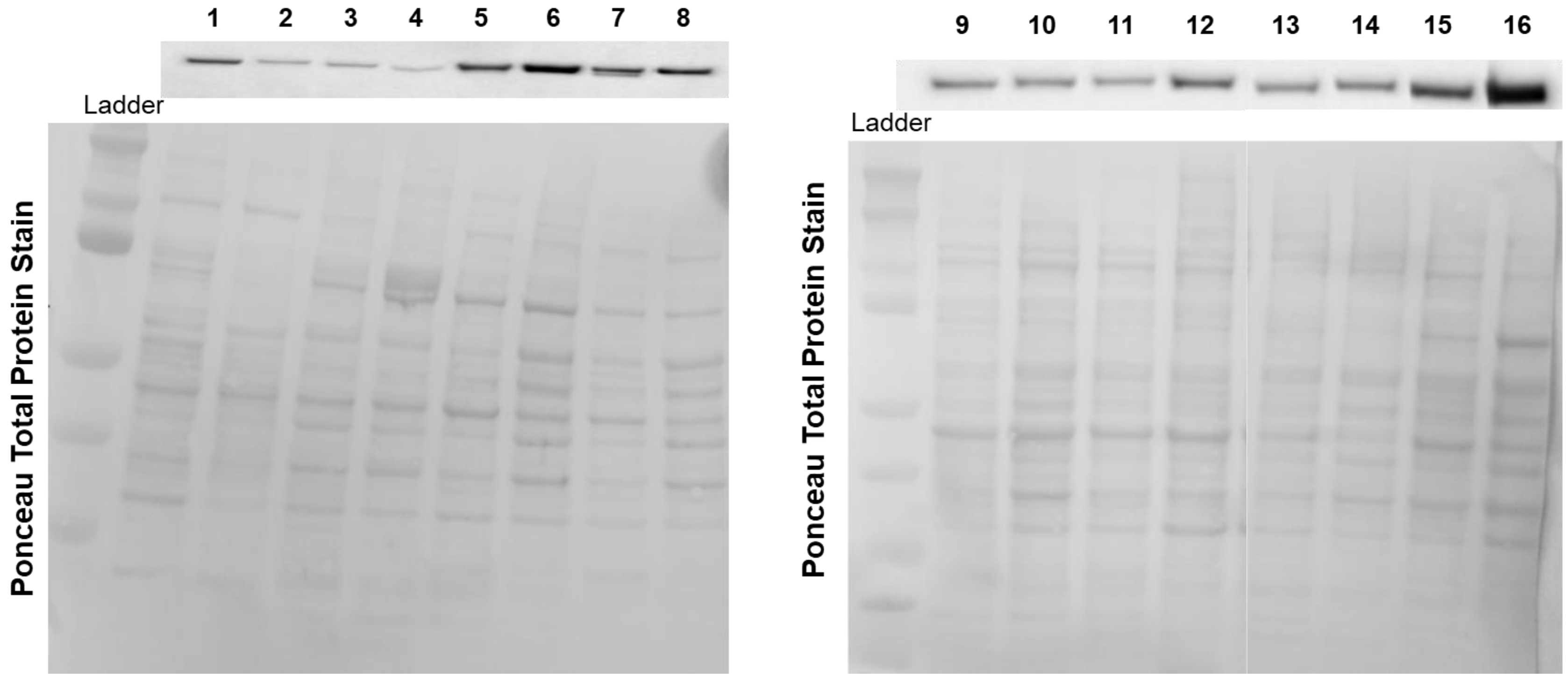

Figure S2. Total protein loading controls for MW2 and C36 mecA swap construct Western Blots. Lanes depicted on Western Blot correspond to ponceau total protein images aligned below. Lanes 1-16 correspond to the following samples: (**1**) MW2 parent (RBS T; 246G) CA-MHB Tris + 1/2X MIC OXA, (**2**) MW2 parent (RBS T; 246G) CA-MHB Tris + 44 mM $NaHCO_3$ + 1/2X MIC OXA, (**3**) MW2 ALC9188 (RBS T; 246E) CA-MHB Tris + 1/2X MIC OXA, (**4**) MW2 ALC9188 (RBS T; 246E) CA-MHB Tris + 44 mM $NaHCO_3$ + 1/2X MIC OXA, (**5**) MW2 ALC9196 (RBS G; 246E) CA-MHB Tris + 1/2X MIC OXA, (**6**) MW2 ALC9196 (RBS G; 246E) CA-MHB Tris + 44 mM $NaHCO_3$ + 1/2X MIC OXA, (**7**) MW2 ALC9200 (RBS G; 246G) CA-MHB Tris + 1/2X MIC OXA, (**8**) MW2 ALC9200 (RBS G; 246G) CA-MHB Tris + 44 mM $NaHCO_3$ + 1/2X MIC

OXA, (**9**) C36 ALC9268 (RBS T; 246G) CA-MHB Tris + 1/2X MIC OXA, (**10**) C36 ALC9268 (RBS T; 246G) CA-MHB Tris + 44 mM $NaHCO_3$ + 1/2X MIC OXA, (**11**) C36 ALC9322 (RBS T; 246E) CA-MHB Tris + 1/2X MIC OXA, (**12**) C36 ALC9322 (RBS T; 246E) CA-MHB Tris + 44 mM $NaHCO_3$ + 1/2X MIC OXA, (**13**) C36 ALC9259 (RBS G; 246E) CA-MHB Tris + 1/2X MIC OXA, (**14**) C36 ALC9259 (RBS G; 246E) CA-MHB Tris + 44 mM $NaHCO_3$ + 1/2X MIC OXA, (**15**) C36 parent (RBS G; 246G) CA-MHB Tris + 1/2X MIC OXA, (**16**) C36 parent (RBS G; 246G) CA-MHB Tris + 44 mM $NaHCO_3$ + 1/2X MIC OXA.

Table S1. Primers used in this study

Primer Name	Sequence (5' to 3')	Application
725	ACTACATTTGTAATATACTACAAATGTAGTCTTATATAAGGAGTATATTGATGATTA AAGGAGAAGAACTTTTCACTGGA	Cloning
726	ATTTATGTCAAAATAATGTTATAATTTTTGTGATATGGAGGTGTAGACGGGTACCG AGCTCGAATTCA	Cloning
727	ACTACATTTGTAATATACTACAAATGTAGTCTTATATAAGGAGGATATTGATGATTA AAGGAGAAGAACTTTTCACTGGA	Cloning
728	ACTACATTTGTAATATACTACAAATGTAGTCTTATATAAGGAGTATATTGATGAAAT TTGGAAACTTTTTGCTTACATACCAA	Cloning
729	ACTACATTTGTAATATACTACAAATGTAGTCTTATATAAGGAGGATATTGATGAAAT TTGGAAACTTTTTGCTTACATACCAA	Cloning
730	AAGAGAAGGATCCTGAGCCATAATCATTTTTCATGTT	Cloning
731	AAGAGAAGGATCCATCGTTACGGATTGCTTCACTGTT	Cloning
732	AAGAGAAGGATCCTTAACTGTTTTAGTTAACTCATGT	Cloning
733	AACCAAATCTTATGTGACATAATATTT	PCR verification
734	GTAATCTGGAACTTGTTGAGCAGA	PCR verification
745 (*mecA* E246G fwd)	TTC CTA GAG GAT AGT TAC GAC TTT CTG	Mutagenesis AA246
746 (*mecA* E246E fwd)	TTT CTA GAG GAT AGT TAC GAC TTT CTG	Mutagenesis AA246
747 (*mecA* E246E rev)	AAG CGA CTT CAC ATC TAT TAG GTT A	Mutagenesis AA246
mecA Fwd	TCCAGATTACAACTTCACCAGG	qRT-PCR
mecA Rev	CCACTTCATATCTTGTAACG	qRT-PCR
gyrB Fwd	CGCAGGCGATTTTACCATTA	qRT-PCR
gyrB Rev	GCTTTCGCTAGATCAAAGTCG	qRT-PCR
